# Exogenous α-amylase improves the digestibility of corn and corn–soybean meal diets for broilers

**DOI:** 10.1016/j.psj.2021.101019

**Published:** 2021-01-27

**Authors:** V.G. Schramm, A. Massuquetto, L.S. Bassi, V.A.B. Zavelinski, J.O.B. Sorbara, A.J. Cowieson, A.P. Félix, A. Maiorka

**Affiliations:** ∗ Department of Animal Science, Federal University of Paraná, Curitiba, Brazil 80035-050; † DSM Nutritional Products, São Paulo, Brazil 00532-110; ‡ DSM Nutritional Products, 4303 Kaiseraugst, Switzerland

**Keywords:** amylase, broiler, corn, starch digestibility, resistant starch

## Abstract

Starch is the main energy source in broiler diets. However, endogenous amylase secretion in young broilers is suboptimal to completely digest dietary starch, so exogenous α-amylase supplementation may help increase starch digestibility. The objective of this study was to assess the supplementation of increasing doses of an exogenous α-amylase (0, 40, 80, 120, and 160 kilo-novo α-amylase units (**KNU**)/kg) on corn and on a complete corn–soybean meal diet for 25-day-old broilers. Jejunal and ileal apparent digestibility coefficients of available starch, resistant starch, total starch, and DM, DM total tract retention, as well as dietary AME levels were evaluated. Interactions (*P* < 0.05) between diets and α-amylase showed that the enzyme had a more evident effect on increasing DM jejunal digestibility and AME on corn compared with the complete diet. Corn DM digestibility increased to a maximum of 67.84% with up to 47 KNU/kg, whereas 89 KNU/kg led to a maximum of 53.92% in the complete diet A maximum increase of 64 kcal AME/kg was obtained with 80 KNU/kg on the complete diet, whereas 109 KNU/kg generated 327 kcal AME/kg on corn (*P* < 0.05). Increasing the α-amylase dose linearly increased ileal digestibility of resistant starch (*P* < 0.05), and the effect on DM total tract retention was quadratic (*P* < 0.05). Corn showed a higher digestibility for DM, resistant and total starch, as well as DM total tract retention and AME, compared with the complete diet (*P* < 0.05). Treatments had no influence on available starch. The inclusion of exogenous α-amylase improves starch, DM, and energy utilization of corn-based and corn–soybean meal–based diets for broilers.

## Introduction

One of the main ingredients in broiler chickens diet is corn, which contains approximately 69% starch (Bach Knudsen, 1997) and supplies more than half of the ME requirements of broiler diets (Weurding et al., 2003). However, the efficiency of corn starch utilization by broilers is influenced by the chemical structure of starch, endogenous secretion of enzymes, feed retention time in the gastrointestinal tract (**GIT**), and feed processing (Carré, 2004; Bello-Pérez et al., 2006).

Approximately 2 to 6% of the starch contained in cereal grains is a fraction called resistant starch (**RS**), which is not digested (Weurding et al., 2001), and is eventually fermented by bacteria in the lower intestinal tract–a reaction that generates energy. Although microbial fermentation of feed can provide up to 11% of broilers ME requirements (Annison et al., 1968), it is less efficient than the digestion process by the host (Dierick et al., 1989). According to Weurding et al. (2001), different grains may have similar total tract digestibility values for starch, but its utilization for metabolic functions can be more efficient when digested in the small intestine.

The duodenum and jejunum are the most important GIT fractions for starch digestion and absorption (Riesenfeld et al., 1980; Zimonja and Svihus, 2009). According to Riesenfeld et al. (1980), the duodenum was the main site of starch degradation and glucose absorption, and most of the digestion products are completed at the end of the jejunum. These dynamics of starch digestion and absorption are influenced by age. Young broilers are less efficient to digest starch because of their limited secretion of endogenous enzymes, which potentially reduces the energy obtained from the diet (Sklan et al., 2003). Studies report that amylase secretion in the duodenum per g of feed intake is low in 4-day-old broilers but increases from 7 and until 21 d of age (Noy and Sklan, 1995; Uni et al., 1995). Apparently, pancreatic amylase secretion may be inadequate in relation to the demands imposed by an increasing starch intake (Noy and Sklan, 1995).

The supplementation of exogenous α-amylase increases starch digestibility, dietary AME content (Isaksen et al., 2011; Stefanello et al., 2015, 2017, 2019; Schramm et al., 2016), and improves broiler's growth performance (Gracia et al., 2003; Onderci et al., 2006; Jiang et al., 2008; Kaczmarek et al., 2014; Stefanello et al. 2015, 2017; Yuan et al., 2017). A better starch digestibility provided by α-amylase supplementation may have positive effects on the physiology of the GIT (Jiang et al., 2008; Yuan et al., 2017), for example reduced secretion of endogenous amylase and reduced pancreatic mass (Gracia et al., 2003; Cowieson et al., 2019), which may spare some amino acids as pancreatic amylase is composed of 16–17% Gly + Ser (Croom et al., 1999).

The positive effects of α-amylase supplementation to broiler diets are evidenced in the literature, but there is a lack of information on how the inclusion of a monocomponent α-amylase affects digestibility of the different starch fractions of corn. Therefore, the objective of this study was to evaluate the effect of increasing supplemented doses of an exogenous α-amylase on jejunal and ileal apparent digestibility of starch fractions, DM utilization, and AME of corn and complete broiler diets based on corn and soybean meal (**SBM**).

## Materials and methods

The experimental procedures were approved by the Committee of Ethics on Animal Use of the sector of Agricultural Sciences of the Federal University of Paraná under the protocol number 035/2012.

### Birds and Facilities

Male Cobb broilers (n = 630) were reared from 1 to 25 d. The experiment was carried out in the metabolism room of the Agricultural Sciences Sector, UFPR, Brazil. The birds were housed in metabolic cages (7 birds per cage) made of galvanized wire (0.90-m long × 0.40-m wide × 0.30-m high) and equipped with trough feeders and drinkers. Metal trays, lined with plastic canvas, were placed under the cages for excreta collection.

Continuous incandescent light (24 h) was supplied during the first 5 d, after which a lighting program of 14L:10D was applied. Room temperature was recorded twice daily using thermometers and was controlled using brooders, incandescent lamps, and opening the windows. On day 1, room temperature was set to 32°C and was gradually reduced by 0.5°C per day until 20°C on day 25. Birds were offered feed and water ad libitum during the entire experimental period.

### Experimental Design and Dietary Treatments

A completely randomized experimental design in a 5 × 2 factorial arrangement was applied. Treatments consisted of 5 α-amylase inclusion levels: 0, 40, 80, 120, or 160 kilo-novo α-amylase units (**KNU**)/kg; and 2 diets: a complete diet based on corn and SBM and a complete diet with 40% replacement for corn; totaling 10 treatments with 9 replicates of 7 birds each. All birds received a standard corn–SBM diet from day 1 to 14 (3,100 kcal/kg AME, 22% CP, 0.9% Ca, and 0.45% available P), and from 15 to 25 d, the experimental diets (Table 1) were fed.

**Table 1:** Feedstuffs and calculated nutritional composition of the experimental diets.

Item	Corn + soybean meal	Corn + soybean meal +40% corn
Feedstuffs		
Corn (%)	56.35	73.40
Soybean meal (%)	34.35	20.61
Soybean oil (%)	4.52	2.712
Phosphate^1^ (%)	1.84	1.104
Limestone (%)	0.91	0.546
Salt (%)	0.48	0.288
L-lysine (%)	0.150	0.090
L-methionine (%)	0.162	0.097
L-threonine (%)	0.040	0.024
Choline chloride (%)	0.050	0.030
Celite^2^ (%)	1.000	1.000
Mineral premix^3^ (%)	0.050	0.030
Vitamin premix^4^ (%)	0.100	0.060
Calculated nutritional composition		
ME (kcal/kg)	3,100	3,200
Sodium (%)	0.220	0.136
Digestible lysine (%)	1.150	0.766
Digestible methionine (%)	0.462	0.334
Digestible Met + Cys (%)	0.759	0.571
Digestible tryptophan (%)	0.232	0.158
Digestible threonine (%)	0.747	0.543
Digestible arginine (%)	1.323	0.922
Chlorine (mg)	1.51	1.08
Electrolyte balance (meq)	199	145
Analyzed nutritional composition		
DM (%)	10.44	10.17
Total starch (%)	46.08	58.72
CP (%)	21.06	15.62
Calcium (%)	0.897	0.560
Total phosphorus (%)	0.701	0.528

1 Composition: 0.013% Cl; 24.00% Ca; 18.5% avP; 0.1 K; 0.06 Na.

2 Indigestible marker (Celite; Celite Corp., Lompoc, CA).

3 Content per kg: iodine, 2 mg; selenium, 200 mg; copper, 20 mg; iron, 50 mg; manganese, 120 mg; zinc, 100 mg.

4 Supplementation per kg of diet: vitamin A, 15,000 IU; vitamin D3, 5,000 IU; vitamin E, 100 mg; vitamin K, 5 mg; folic acid, 3 mg; nicotinic acid, 75 mg; pantothenic acid, 25 mg; riboflavin, 8 mg; thiamine, 5 mg; pyridoxine, 7 mg; biotin, 300 mg; choline, 400 mg; vit. B12, 20 mg.

The partial substitution method was used to calculate the coefficients of digestibility on corn, where 60% of the diet was composed by the complete corn–SBM diet, and the remaining 40% was replaced by corn as per the methodology by Matterson et al., 1965. The diet with 40% replacement for corn was used as a test diet, and this substitution was performed to extrapolate the diet to 100% corn and evaluate the effects on corn digestibility irrespectively of the other ingredients. The analyzed corn composition is shown in Table 2. After replacement, the same 5 increasing doses of α-amylase applied to the experimental diets were added to the corn-based test diet.

**Table 2:** Chemical nutritional composition of corn used in the experiment.

Nutrient	DM (%)
DM	87.05
CP	7.45
Total fiber^1^	8.65
Insoluble fiber^1^	7.55
Soluble fiber^1^	1.10
Total starch	78.00
Amylose in corn^2^	18.74
Amylose in starch^2^	24.00
Ash	1.00
Calcium	0.03
Phosphorus	0.22

1 Method proposed by Prosky et al. (1988).

2 Amylose content obtained by the Blue Value method (Gilbert and Spragg, 1964) using hydrolyzing enzymes: α-amylase (Termamyl 120 Ls), protease (Flavorourmeme 500 Ls), and amyloglucosidase (AMG 300 Ls), all produced by Novozymes Latin American Limited.

The α-amylase product (RONOZYME HiStarch CT; Novozymes A/S, Bagsvaerd, Denmark) is a heat-tolerant enzyme containing α-amylase (IUB No. 3.2.1.1) produced by the fermentation of a genetically modified microorganism (*Bacillus licheniformis*), with a minimum activity of 600 KNU/g. One kilo novo α-amylase unit is the amount of enzyme that releases 5.26 g of starch per h in a two-step reaction, 6 μmol p-nitrophenol per min from 1.86 mM ethyledene-G7-p-nitrophenyl-maltoheptaoside at pH 7.0 and 37°C. The enzyme was mixed with 1 kg of corn before being added to the diet mixing. α-Amylase activity in the experimental diets is presented in Table 3.

**Table 3:** Declared and analyzed α-amylase activity in the experimental diets.

Diets	Amylase, KNU^1^/kg	
	Declared	Analyzed
Corn + Soybean meal	0	<LOD
Corn + Soybean meal	40	32
Corn + Soybean meal	80	53
Corn + Soybean meal	120	70
Corn + Soybean meal	160	141
Corn + Soybean Meal +40% Corn	0	<LOD
Corn + Soybean Meal +40% Corn	40	44
Corn + Soybean Meal +40% Corn	80	65
Corn + Soybean Meal +40% Corn	120	127
Corn + Soybean Meal +40% Corn	160	151

Abbreviation: LOD = limit of detection.

1 Kilo novo α-amylase units is the amount of enzyme that releases 5.26 g of starch per h in a two-step reaction, 6 μmol p-nitrophenol per min from 1.86 mM ethyledene-G7-p-nitrophenyl-maltoheptaoside at pH 7.0 and 37°C.

### Growth Performance

At the beginning of the experiment (day 15), all birds were weighted into groups of 7 birds before being allocated in the metabolic cages, and the average initial BW per group was 504.5 g. At day 15 and 25, all birds were weighted, averaged by cage. Feed intake, BW gain (**BWG**), and feed conversion ratio (**FCR**) corrected to the weight of dead birds from 15 to 25 d were calculated.

### Digestibility Assay

Birds were submitted to a 5-d period of adaptation to the experimental diets (Day 15–20), after which excreta samples were collected using the partial collection method for 4 d (Day 21–24). Excreta were collected twice daily, with the aid of plastic spatulas, placed in duly identified plastic bags immediately after collection, and then frozen at −18°C.

To determine starch and DM digestibility in the jejunal and ileal contents, all birds were euthanized on day 25 by cervical dislocation and eviscerated, and the intestinal tract content was collected. The jejunal fraction was defined as 4 cm after the end of the pancreas and 4 cm above the Meckel diverticulum, and the ileal fraction defined as 4 cm below the Meckel diverticulum and 4 cm above the ileo-ceco-colonic junction. The jejunal and ileal contents were manually removed by compressing these segments with the aid of scissors and forceps, placed in duly identified plastic recipients, immediately frozen in liquid nitrogen, and stored in a freezer at −18°C.

### Chemical Analyses

Excreta samples were thawed at room temperature and homogenized. Aliquots were dried in an oven at 55°C until constant weight. Jejunal and ileal contents were frozen at −20°C and subsequently freeze-dried (Modulyo D Freeze Drier; Thermo Electron Co., Waltham, MA) at a vacuum pressure of 5 × 10^−2^ m bar and ground to 1-mm particle size. DM content of diets, corn, jejunal digesta, and ileal digesta was determined by drying samples to a constant weight in an oven at 105°C, as per the Association of the Official Analytical Chemists (AOAC, 2000). Gross energy of the excreta and diets was determined in a bomb calorimeter (IKA model 1261; Parr Instrument Co., Moline, IL). Acid insoluble ash was included at 1% in the experimental diets as an indigestible marker, and acid insoluble ash content in the diets, excreta, jejunal, and ileal contents was determined as per methodology by Scott and Boldaji (1997). CP (method 954.01), ash (method 942.05), calcium (method 927.02), and total phosphorus (method 965.17) content of the diets and corn were analyzed as per the Association of the Official Analytical Chemists (AOAC (2000).

Total starch (**TS**), available starch (**AvS**), and RS levels in the diets and jejunal and ileal contents were analyzed aas per the Association of the Official Analytical Chemists method 996.11, adapted by Walter et al. (2005). In this method, samples (100 mg) are incubated in a solution with amylase for 5 min and amyloglucosidase for 30 min, after which the supernatant is considered AvS. The residue is then treated with dimethyl sulfoxide; a new incubation with amylase and amyloglucosidase is conducted for the same amount of time; and the supernatant is then quantified as RS. Both AvS and RS quantities add up to TS.

Digestibility of starch fractions and DM, total tract retention (**TTR**) of DM, and AME on the complete diet were calculated as per equations by Sakomura and Rostagno (2016) and using the indigestibility factor (**IF**):

$$IF\;=\;\frac{\%\;AIA\;in\;the\;diet}{\%\;AIA\;in\;the\;excreta.}$$

DM TTR was calculated as follows:

DMTTR=100–IF.

AME was calculated as follows:

AME=dietaryGE–(excretaGExIF).

The coefficient of apparent digestibility (**CD**) of AvS, RS, TS, and DM measured in the jejunal and ileal contents (**CDj** and **CDi**, respectively) for the complete diet were calculated as follows:

$$CDji=\frac{\left[Nutrient\;dietary\;content–\left(Nutrient\;intestinal\;content\;x\;IF\right)\right]}{Nutrient\;dietary\;content\;x\;100.}$$

Corn digestibility, DM TTR, and AME were calculated using the equation proposed by Matterson et al. (1965). An example of the equation for CDij goes as follows (the same equation is used to determine DM TTR and AME on corn):

$$CDij\;Corn=CDij\;of\;complete\;diet+\left[\frac{CDij\;of\;diet\;with\;40\%\;corn-CDij\;of\;complete\;diet}{\left(\%\;corn\;inclusion\;*\;corn\;DM\;content\right)}\right]$$

### Statistical Analyses

Data normality was analyzed by the Shapiro-Wilk test, and data with normal distribution were submitted to ANOVA at 5% significance level. When the interaction between factors were significant, means were compared by Tukey's test at 5% probability level. When the effect of increasing levels of α-amylase inclusion on the analyzed variables was significant, data were submitted to linear and quadratic analysis of regression. Maximum response of the evaluated variable to α-amylase supplementation was determined using linear response plateau (Robbins, 1986) analysis.

## Results and discussion

No interaction between diets and α-amylase was observed for Feed intake, BWG, and FCR (Table 4). Feed intake was not affected by the dietary treatments, but broilers fed the diet with partial substitution of corn had lower BWG and worse FCR. This was expected, as the substitution of 40% of the diet with corn inevitably resulted in lower dietary levels of CP, amino acids, and macrominerals compared with the complete diet, which then limited the broilers' growth. These results agree with Stefanello et al. (2019), who assessed the same partial substitution method proposed by Matterson et al. (1965) and reported lower BWG and worse FCR on broilers fed corn–SBM diets displaced with 40% corn.

**Table 4:** Growth performance of broilers fed complete corn–soybean meal based or complete diets substituted with 40% corn supplemented with α-amylase from 15 to 25 d of age.

Diets	Α-Amylase, KNU^1^/kg	FI^2^ (g)	BW^3^ (g)	FCR^4^ (g)
Effect of interaction				
Corn and soybean meal diet	0	1,115	740	1.509
	40	1,074	710	1.515
	80	1,117	738	1.516
	120	1,075	713	1.509
	160	1,103	754	1.464
Corn-based	0	1,072	579	1.860
	40	1,157	610	1.918
	80	1,132	590	1.933
	120	1,091	605	1.808
	160	1,094	580	1.903
SEM		7.23	9.11	0.025
Effect of diet				
Corn and soybean meal diet		1,097	731	1.503
Corn-based		1,109	593	1.884
Effect of α-amylase inclusion				
	0	1,094	660	1.685
	40	1,116	660	1.717
	80	1,125	664	1.725
	120	1,083	659	1.659
	160	1,099	667	1.684
Probabilities				
Diet		0.630	<0.001	<0.001
Amylase		0.651	0.720	0.567
Diet∗Amylase		0.755	0.685	0.726

1 Kilo novo α-amylase units.

2 Feed intake.

3 BW gain.

4 Feed conversion ratio.

**Figure 1:**
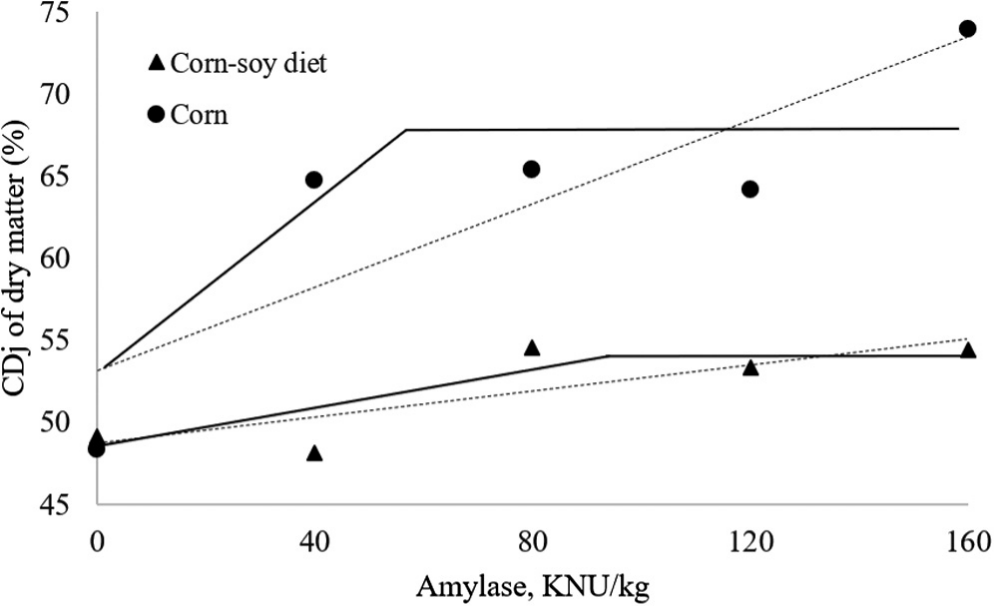
Effect of increasing α-amylase doses on the coefficient of apparent jejunal digestibility (CDj) of DM of corn (*P*-linear<0.001, y = 0.126x + 53.19, R^2^ = 0.74; response linear plateau: break point=67.84%, optimal dose=47 KNU/kg) and a complete corn–soy diet (*P*-linear = 0.005, y = 0.039x + 48.75, R^2^ = 0.67; response linear plateau: break point=53.92%, optimal dose=89 KNU/kg) for 25-day-old broilers. Abbreviation: KNU, Kilo novo α-amylase unit.

The obtained CDj and CDi of DM, AvS, RS, and TS, DM TTR, and AME (kcal/kg DM) are presented in Table 5. An interaction between diet and α-amylase levels was detected for DM CDj (*P* < 0.05). The increase in DM jejunal digestibility as a function of amylase concentration was more evident for corn compared with the standard corn–SBM diet, which is further highlighted in Figure 1. This effect is presumably associated with a greater substrate concentration in corn than in the complete diet. When comparing the treatments without α-amylase with those with the highest dose (160 KNU/kg), the CDj of DM increased by 50% in corn, whereas an increase of 10% was obtained in the complete diet. The linear response plateau analysis shows that the optimal dose of α-amylase in the complete diets was 89 KNU/kg, resulting in a DM CDj of 53.92%. In corn, however, the optimal dose was 47 KNU/kg, increasing DM CDj up to 67.84%. Corn starch granules are embedded in a protein matrix (Watson, 1987), so an increase on starch digestibility is accompanied by a greater release of protein for endogenous enzyme digestion. In addition, starch granules in cereals contain approximately 1 to 14 g lipids/kg (Buleon et al., 1998; Abdel-Aal et al., 2002) and 3 g protein/kg (Cornell et al., 1994; Hoover and Vasanthan, 1994; Vasanthan and Bhatty, 1996; Abdel-Aal et al., 2002), so up to 1.7% of nutrients other than starch may be encapsulated in the starch granule. The supplementation of α-amylase may have successfully released those nutrients from the starch granules, which contributed to the increase in CDj values of DM for both corn and complete diets.

**Table 5:** Jejunal and ileal coefficients of apparent digestibility of DM, available starch, resistant starch, total starch, total DM retention in the tract, and AME of an extrapolated 100% corn and a complete corn–soybean meal diet supplemented with α-amylase in 25-day-old broilers.

Diet	Α-amylase, KNU^1^/kg	CDj^2^				CDi^3^				RTT^7^	AME (kcal/kg DM)
		DM	AvS^4^	RS^5^	TS^6^	DM	AvS	RS	TS	DM	
Effect of interaction											
Corn and soybean meal diet	0	49.14^c^	78.62	60.43	77.38	74.00	96.12	78.01	95.11	74.22	3,711^b^
	40	48.13^c^	78.98	65.15	77.49	73.81	95.42	80.19	94.37	75.11	3,736^b^
	80	54.53^b,c^	77.48	64.94	76.34	74.60	96.62	80.28	95.71	75.88	3,775^b^
	120	53.34^b,c^	77.60	65.15	74.01	74.48	96.71	85.94	96.08	78.84	3,779^b^
	160	54.43^b,c^	80.45	64.75	79.93	74.29	95.06	83.59	94.33	76.14	3,762^b^
Corn	0	48.33^c^	82.67	83.55	85.18	82.25	95.60	86.87	96.39	87.06	3,696^b^
	40	64.78^a,b^	85.29	80.02	88.31	81.20	95.46	88.44	95.08	88.29	3,770^b^
	80	65.40^a,b^	86.97	88.56	87.08	83.40	96.29	91.50	96.47	90.83	3,946^a^
	120	64.18^a,b^	87.67	90.82	88.40	85.27	97.52	94.36	96.38	91.84	4,019^a^
	160	73.96^a^	86.13	90.04	86.58	85.45	94.92	95.44	93.11	91.41	4,028^a^
SEM		1.20	0.92	0.91	2.02	0.61	0.25	0.30	0.88	0.78	16.22
Effect of diet											
Corn and soybean meal diet		51.91	78.63	64.08	77.03	74.24	95.99	81.60	95.12	76.04	3,753
Corn-based		63.33	85.75	86.60	87.11	83.51	95.96	91.32	95.49	89.89	3,892
Effect of α-amylase inclusion											
	0	48.74	80.65	71.99	81.28	78.13	95.86	82.44	95.75	80.64	3,704
	40	56.46	82.14	72.59	82.90	77.51	95.44	84.32	94.73	81.70	3,753
	80	59.97	82.23	76.75	81.71	79.00	96.46	85.89	96.09	83.36	3,861
	120	58.76	82.64	77.99	81.21	79.88	97.12	90.15	96.23	85.34	3,899
	160	64.20	83.29	77.40	83.26	79.87	94.99	89.52	93.72	83.78	3,895
Probabilities											
Diets		<0.001	<0.001	<0.001	<0.001	<0.001	0.106	<0.001	0.008	<0.001	<0.001
Amylase		<0.001	0.796	0.767	0.966	0.175	0.294	<0.001	0.238	<0.001	<0.001
Diet∗Amylase		0.009	0.711	0.822	0.392	0.569	0.854	0.519	0.060	0.431	<0.001

Means followed by different superscripts in the same column are significantly different.

1 Kilo novo α-amylase units.

2 Coefficients of apparent digestibility in the jejunal content of DM.

3 Coefficients of apparent digestibility in the ileal content of DM.

4 Available starch.

5 Resistant starch.

6 Total starch.

7 DM retention in the total tract.

In the present study, the complete corn–SBM diet differed from corn regarding all the analyzed variables (*P* < 0.05), with an exception for CDi AvS. Corn starch digestibility is greater (>96%) than other cereals commonly used in animal nutrition (Carré, 2004; Meng and Solominski, 2005). In 21-day-old chickens, Svihus et al. (2004) determined a 97% starch digestibility of corn in the ileum and Skiba et al. (2003) in an experiment with 20- to 24-day-old broilers determined 91 to 96% corn starch digestibility. Comparing different cereals, Svihus (2001) obtained 79, 96, and 99% starch digestibility in wheat, barley, and oats, respectively. Although most published studies state that corn starch digestibility is high, some authors claim that it rarely exceeds 85% (Noy and Sklan, 1995). This difference can be related to the age of broilers at the time of evaluation. Zelenka and Ceresnakova (2005) evaluated the effects of age on starch digestibility of broiler chickens and observed a linear increase of 4.4% on starch digestibility between day 1 and 22 of age, and at 22 d, starch digestibility reached its maximum value (99%) which reflects a better capacity for synthesizing endogenous amylase on older broilers. Differences in the methods of starch digestibility analysis may also influence the results, as well as the intrinsic differences in corn and starch previously mentioned. In the present study, starch digestibility ranged from 85 to 88% in the jejunum and 93 to 96% in the ileum. These results are consistent with the findings of Riesenfeld et al. (1980), who reported 65% starch digestibility in the duodenum, 85% in the jejunum, and about 97% in the final segment of the ileum.

Starch CDj, as well as CDi of DM, AvS, and TS, were not influenced by α-amylase inclusion or by an interaction between the enzyme and diets. The results differ from those of the study by Aderibigbe et al. (2020), who observed a linear increase in starch digestibility at the posterior jejunum when supplementing increasing doses of α-amylase (0, 80 and 160 KNU/kg) to a corn–SBM diet.

No differences in AvS CDi values were observed between diets or α-amylase doses. The AvS portion of starch is considered easily digestible by the animal, whereas RS is resistant to enzymatic digestion in the small intestine (Weurding et al., 2003; Walter et al., 2005). Therefore, the lack of statistical differences in AvS CDi may have been due to the fact that AvS digestibility was naturally high in both diets and was rapidly digested before reaching the ileum, so the inclusion of exogenous α-amylase did not promote any further improvements.

α-Amylase supplementation had isolated effects on RS CDi (*P* < 0.05). Increasing α-amylase levels promoted a linear response of RS CDi (*P* = 0.024; y = 0.05x + 82.46; R^2^ = 0.91). To be used as a feedstuff, corn may previously be subjected to a drying process with high temperatures, which can impact the grain's nutritional composition. According to Penfield and Campbell (1990), the optimal drying temperature for corn to minimize nutritional losses ranges from 62°C to 75°C. In Brazil, corn is usually dried at much higher temperatures, around 80°C to 120°C. Although starch is gelatinized at high drying temperatures, the immediate drop on the temperature of the grain after drying causes starch to undergo a reverse process of gelatinization, called retrogradation (Atwell et al., 1988). Retrogradation consists on the reorganization of amylose chains – which are linked by hydrogen bonds – into helical pairs (Bello-Pérez et al., 2006). Water is removed from inside the starch granule, increasing its viscosity (Lajolo and Menezes, 2006) and further limiting the enzymatic action and digestion (Englyst et al., 1992; Muir e O'dea, 1992), leading to the formation of retrograded or type 3 RS. However, the supplementation of exogenous α-amylase may reduce the negative effects of high-temperature drying of corn and improve retention of starch by the host, as seen in the present study. It is also important to mention that an increase in starch digestibility, especially RS, results in less starch reaching the distal gut, and consequently, there is less substrate to be fermented by the microbiota (Weurding et al., 2003). Considering this notion, broilers' diet supplementation with amylases or other carbohydrases will typically affect the GIT microbiota, as evidenced in the literature (Weurding et al., 2003; Yin et al., 2018; Craig et al., 2020).

α-Amylase supplementation influenced DM TTR (*P* < 0.05). Increasing the enzyme's doses had a quadratic effect on DM TTR (*P* = 0.048; y = −0.0002 × 2 + 0.059x + 80.27; R^2^ = 0.85). These results are in agreement with those of the study by Zanella et al. (1999), who found greater starch ileal digestibility (from 91.2 to 93.0%) and starch TTR (from 98.2 to 98.5%) when broilers were fed an enzyme blend containing amylase, protease, and xylanase compared with unsupplemented diets. In addition, Zhang et al. (2012) observed a quadratic nutrient digestibility and energy efficiency responses to multienzymatic levels in corn. According to Caspary (1992), high starch digestibility also leads to an increase on intestinal absorption surface area, improving the digestibility of dietary fractions other than starch, and this may explain the observed effects on DM TTR on the present study.

**Figure 2:**
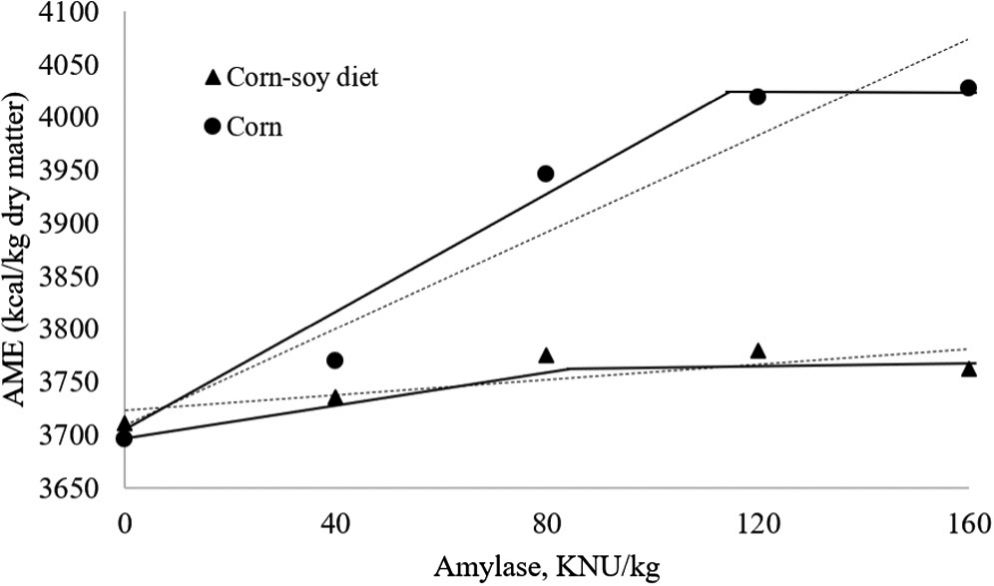
Effect of increasing amylase doses on AME of corn (*P*-linear<0.001, y = 2.28x + 3,709, R^2^ = 0.92; response linear plateau: break point=4,023 kcal, optimal dose=109.6 KNU/kg) and of a complete corn–soy diet (*P*-linear = 0.016, y = 0.362x + 3,723, R^2^ = 0.64; response linear plateau: break point=3,771 kcal, optimal dose=80 KNU/kg) for 25-day-old broilers. Abbreviation: KNU, Kilo novo α-amylase unit.

An interaction was observed between diet type and α-amylase levels for AME (*P* < 0.05). Greater AME results were obtained for corn, and the optimal level of α-amylase inclusion was 80 and 109 KNU/kg, which generated 60 and 327 kcal AME/kg for corn–SBM–based diets and corn (Figure 2),respectively. Other studies also report the effects of α-amylase on increasing AME of complete corn–SBM diets, as Gracia et al. (2003) observed raises on AME of 1.7 and 3% in 21- and 42-day-old broilers, respectively, and Stefanello et al. (2015) a 2% AME increase in 25-day-old broilers, similar to our results. Other studies showed AME improvements of 2.0 to 2.9% in corn–SBM diets supplemented with enzyme blends containing α-amylase (Zanella et al., 1999; Douglas et al., 2000; Rutherfurd et al., 2007; Stefanello et al., 2015). Stefanello et al. (2019) reported a 4% improvement in corn AME with increasing α-amylase doses (from 0 to 160 KNU/kg). An explanation to the interaction could be related to the greater presence of substrate (starch) in corn than in the complete diet. This greater level of starch possibly saturated the capacity of endogenous amylase to process the incoming starch, whereas the activity of the exogenous α-amylase was augmented in comparison with the diet with less substrate.

## Conclusions

The supplementation of exogenous α-amylase improved the digestibility of DM, RS, TTR of DM, and AME for 25-day-old broilers. The effects of increasing α-amylase doses on jejunal digestibility of DM and AME were more evident in corn compared with a complete corn–SBM diet. Supplementing 47 KNU/kg in an extrapolated 100% corn diet increased DM digestibility to a maximum of 67.84%, whereas 89 KNU/kg led to a maximum of 53.92% in the complete diet. Corn AME increased by 327 kcal/kg with up to 109 KNU/kg, whereas an increase of 60 kcal AME/kg was obtained with the inclusion of up to 80 KNU/kg in the complete diet.
